# Gravitational effects on fibroblasts’ function in relation to wound healing

**DOI:** 10.1038/s41526-023-00286-z

**Published:** 2023-06-21

**Authors:** Wilhelmina E. Radstake, Kiran Gautam, Silvana Miranda, Cynthia Van Rompay, Randy Vermeesen, Kevin Tabury, Mieke Verslegers, Alan Dowson, Jeffrey Gorissen, Jack J. W. A. van Loon, Nigel D. L. Savage, Sarah Baatout, Bjorn Baselet

**Affiliations:** 1 Radiobiology Unit, SCK CEN, Belgian Nuclear Research Centre, 2400 Mol, Belgium; 2 Department of Molecular Biotechnology, Faculty of Bioscience Engineering, Ghent University, 9000 Ghent, Belgium; 3 Department of Biomedical Engineering, University of South Carolina, Columbia, SC 29208 USA; 4 Serco Nederland for the European Space Agency (ESA), European Space Research and Technology Centre (ESTEC), TEC-MMG, Keplerlaan 1, 2201 AZ Noordwijk, the Netherlands; 5 ESA/ESTEC, Keplerlaan 1, 2200 AZ Noordwijk, The Netherlands; 6 Department of Oral and Maxillofacial Surgery/Pathology, Amsterdam Movement Sciences & Amsterdam Bone Center (ABC), Amsterdam UMC location Vrije Universiteit Amsterdam & Academic Center for Dentistry Amsterdam (ACTA), Gustav Mahlerlaan 3004, 1081 LA Amsterdam, The Netherlands; 7 HE Space Operations for the European Space Agency, ESA/ESTEC, Keplerlaan 1, 2200 AZ Noordwijk, The Netherlands

**Keywords:** Cell biology, Molecular biology

## Abstract

The spaceflight environment imposes risks for maintaining a healthy skin function as the observed delayed wound healing can contribute to increased risks of infection. To counteract delayed wound healing in space, a better understanding of the fibroblasts’ reaction to altered gravity levels is needed. In this paper, we describe experiments that were carried out at the Large Diameter Centrifuge located in ESA-ESTEC as part of the ESA Academy 2021 Spin Your Thesis! Campaign. We exposed dermal fibroblasts to a set of altered gravity levels, including transitions between simulated microgravity and hypergravity. The addition of the stress hormone cortisol to the cell culture medium was done to account for possible interaction effects of gravity and cortisol exposure. Results show a main impact of cortisol on the secretion of pro-inflammatory cytokines as well as extracellular matrix proteins. Altered gravity mostly induced a delay in cellular migration and changes in mechanosensitive cell structures. Furthermore, 20 × *g* hypergravity transitions induced changes in nuclear morphology. These findings provide insights into the effect of gravity transitions on the fibroblasts’ function related to wound healing, which may be useful for the development of countermeasures.

## Introduction

Deep space is a hostile environment for the Earth-bound human body. Weightlessness, resulting from a state of constant free fall around the Earth, is probably the most evident change in environmental factors. Together with increased psychological stress levels resulting from high workload, social isolation, and living within the confinement of the spacecraft, these spaceflight stressors strongly affect human physiological systems and form a serious threat to the human body in the long term. Problems related to muscular-skeletal deconditioning, microgravity-induced fluid-shift, and cardiovascular issues are among the spaceflight-related disorders^1^.

One of the organs affected during spaceflight is the skin. Shielding the internal body from harmful environmental factors, proper skin function, and hence maintaining a proper barrier function is vital for human health^2^. Astronauts often report skin-related problems, including itches, rashes, and dryness of the skin^3,4^. Multiphoton analyses of astronauts’ skin pre- and post-flight have shown that the space environment can induce skin alterations such as thinning of the epidermis, changes in extracellular matrix (ECM) proteins, loss of skin elasticity and delayed epidermal proliferation and dehydration^5–7^. However, more recent data show improvement in skin hydration and barrier function and no changes in skin density, thickness and elasticity in a larger (*n* = 6) cohort of astronauts^8^. Nevertheless, differences in skin parameters were still observed between astronauts indicating possible individual sensitivity of the skin to the spaceflight environment.

Besides the above-mentioned skin problems observed during and after long-duration spaceflight, small cutaneous injuries as well as signs of delayed wound healing are observed in space^3^. Wound healing is a complex process involving several distinct stages and multiple cellular components. Upon wounding, blood clot formation stops the bleeding and inflammatory cells migrate to the wound site and start clearing the wound from pathogens. During the following proliferating phase, fibroblasts, keratinocytes, and endothelial cells migrate to the site of the wound, start proliferating and remodeling the damaged tissue. These processes continue in the final stage, or remodeling phase, where the wound is further contracted, and scar tissue is formed^9^.

Fibroblasts, which are the major cellular component of the dermis, play a vital role in wound healing by migrating to the side of the wound and interacting with the ECM^9,10^. Earth-based simulation studies have verified the notion of delayed wound healing and showed decreased migration capabilities in fibroblasts after exposure to simulated microgravity^11,12^. Delay in the wound-healing process can also be found during periods of chronic psychological stress^13,14^.

**Fig. 1:**
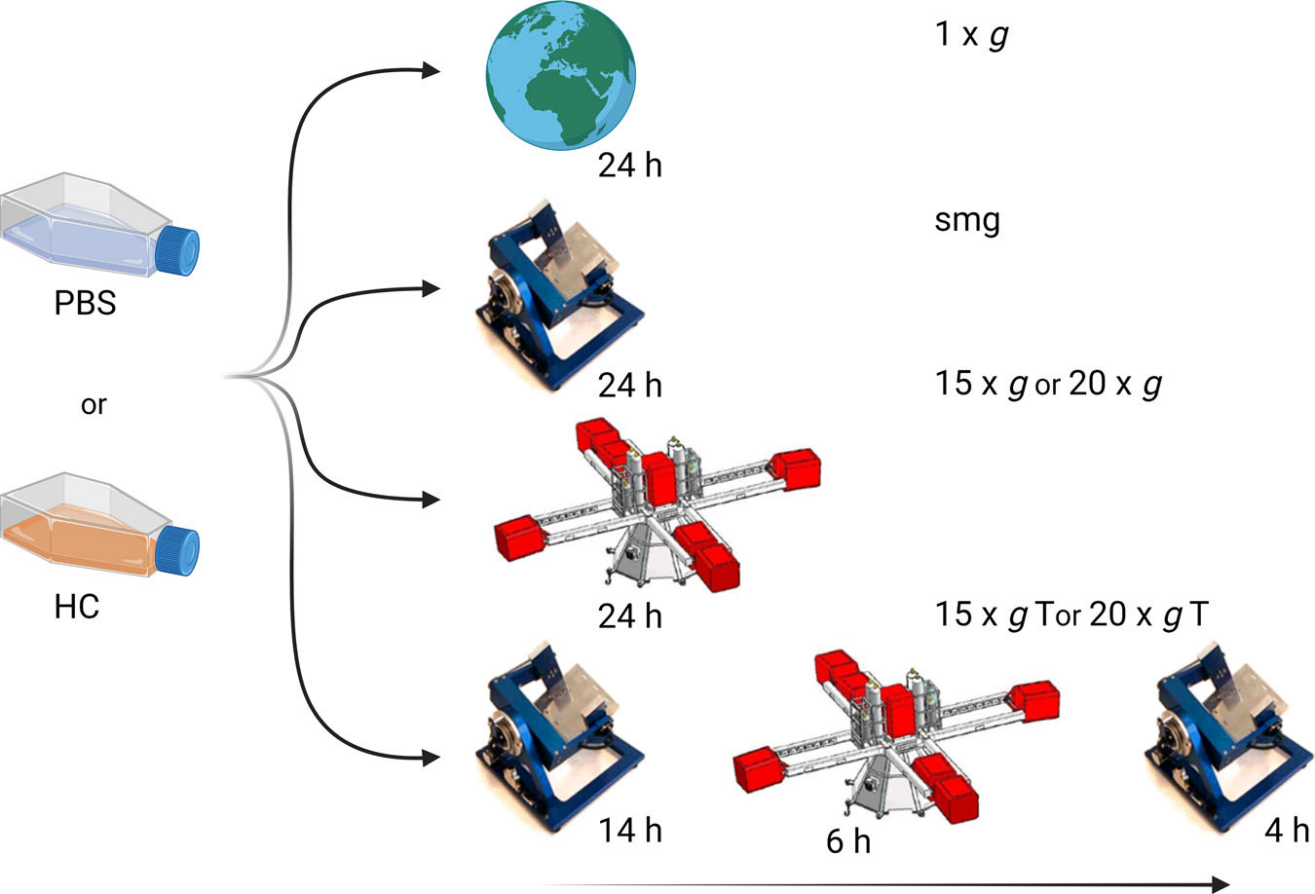
Overview of the experimental setup. Addition of hydrocortisone (HC) or a control vehicle (PBS) to the medium was done 48 h before exposure to altered gravity levels and was followed by exposure to simulated microgravity (smg) by means of the Random Positioning Machine or hypergravity at 15 × *g* or 20 × *g* by means of the Large Diameter Centrifuge (LDC) located at ESA-ESTEC. Two additional groups were exposed to simulated microgravity intermitted with a temporary hypergravity exposure at either 15 × *g* T or 20 × *g* T after which they were placed back into simulated microgravity. Finally, 1 × *g* controls were placed in the center of the LDC. This figure was created with BioRender.com.

From the above, it is clear that the spaceflight environment poses a risk for maintaining healthy skin and for successful healing of skin wounds. In the interest of long-duration interplanetary space missions, there is an increasing urgency to develop effective countermeasures for the observed spaceflight-induced skin changes. Hereto, a better understanding of how dermal fibroblasts react to different gravitational levels and to the induction of stress, together with an investigation of the underlying mechanisms is key. In this study, an in vitro model of scratched cells is used to better understand the impact of altered gravity levels on fibroblasts’ function related to wound healing. As such, wound healing in this paper refers to this in vitro model. After wounding the cells, we exposed normal human dermal fibroblasts (NHDF) to a set of altered gravity levels including simulated microgravity, hypergravity and several gravity transitions between simulated micro- and hypergravity. In addition, we added the stress hormone cortisol to the cell culture medium in order to investigate possible interaction effects between both spaceflight-related stressors (Fig. 1 for an overview of the experimental setup). Several endpoints of fibroblasts’ function related to wound healing, including cytokine expression, migration, cytoskeletal remodeling, and ECM protein expression, were included.

## Results

### Hypergravity significantly delayed fibroblast migration

**Fig. 2:**
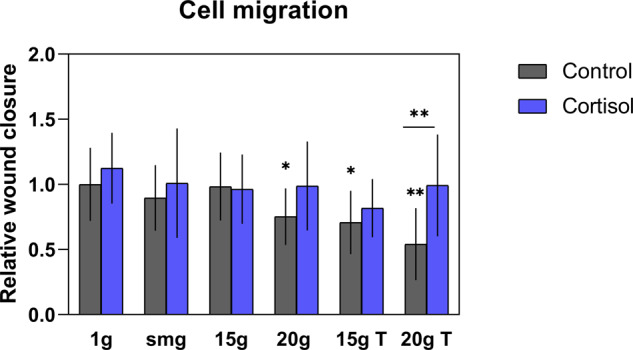
Effect of altered gravity and cortisol exposure on fibroblast migration capacity. Hypergravity exposure to 20 × *g* significantly delayed fibroblast migration compared to 1 × *g* controls (*P* = 0.033, *n* = 10). Simulated microgravity intermitted with 15 × *g* (*P* = 0.016, *n* = 11) and 20 × *g* (*P* = 0.007, *n* = 4) hypergravity also reduced fibroblast migration. The gravity-induced effects were not significant in fibroblasts exposed to cortisol except for the 15 × *g* T group (*P* = 0.0468, *n* = 5). smg = simulated microgravity. 15 ×  *g* T and 20 × *g* T are hypergravity transition groups. Error bars show standard deviations. *P < 0.05, **P < 0.005. Statistical tests included ANOVA followed by post hoc analysis (see “Statistical analyses“). Asterisks without line show significance level compared to 1 × *g* control. Measures are divided by mean value of 1 × *g* controls to obtain relative values.

To test the effect of altered gravity levels and cortisol on the migration capacity of fibroblasts, an in vitro scratch wound assay was performed. Both gravity level (*P* = 0.009, *F* (5, 127) = 3.179, *η*² = 10.2) and cortisol (*P* = 0.003, *F* (1, 127) = 8.954, *η*² = 5.8) exposure significantly influenced migration capacity of fibroblasts. No significant interaction effect was found between both factors (*P* = 0.26, *F* (5, 127) = 1.32). Fibroblasts exposed to 20 × *g* (*P* = 0.033, *n* = 10), 15 × *g* transition (*P* = 0.016, *n* = 11) and 20 × *g* transition (*P* = 0.007, *n* = 4) had a significant delay in wound closure (Fig. 2). However, in fibroblast exposed to cortisol this gravity-induced delay in migration at these gravity levels was not significant.

### Focal adhesions reduced in altered gravity

**Fig. 3:**
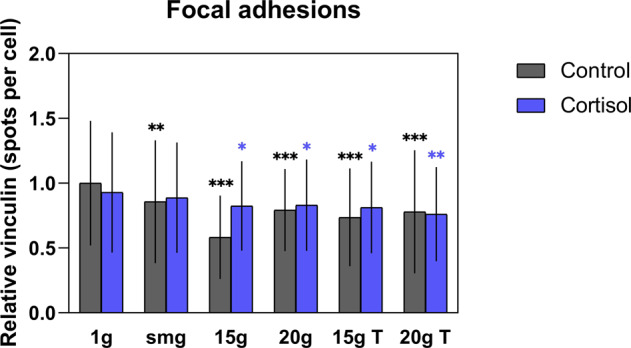
Effect of altered gravity levels and cortisol exposure on the number of vinculin spots per cell. The most evident reduction was found in fibroblasts exposed to 15 × *g* hypergravity and control vehicle. For each group, at least 100 cells were analyzed (with exception of the control vehicle –20 × *g* T group where 84 cells in total were analyzed). smg = simulated microgravity. 15 × *g* T and 20 × *g* T are hypergravity transition groups. Error bars show standard deviations. *P < 0.05, **P < 0.001, ***P < 0.0001. Statistical tests included ANOVA followed by post hoc analysis (see "Statistical analyses”). Asterisks show significance level compared to 1 × *g* control or cortisol exposed cells. Measures are divided by mean value of 1 × *g* controls to obtain relative values.

Focal adhesions connect the actin cytoskeleton with the extracellular matrix through a series of transmembrane integrins and proteins, including vinculin^15^. To investigate the effect of altered gravity levels and cortisol exposure on focal adhesion complexes, the total amount of vinculin spots per cell were quantified. Both altered gravity (*P* < 0.0001, *F* (5, 1646) = 15.21, *η*² = 4.3) and cortisol exposure (*P* = 0.015, *F* (1, 1646) = 5.94, *η*² = 0.3) affected the number of vinculin spots. Moreover, a significant interaction effect (*P* < 0.0001, *F* (5, 1646) = 5.22, *η*² = 1.5) was found between both factors. At all levels, altered gravity reduced the amount of focal adhesion complexes. This was found in fibroblasts exposed to control vehicle as to cortisol. Fibroblasts exposed to control vehicle and 15 × *g* had the lowest count of vinculin spots per cell and significantly differed from other groups. In fibroblasts exposed to both simulated microgravity and cortisol, there was no significant difference observed as compared with cortisol-exposed fibroblasts at 1 × *g*. Furthermore, a significant (*P* < 0.0001, *n* = 166) increase in the amount of focal adhesions was found in fibroblasts exposed to cortisol at 15 × *g* as compared with the control group at the same gravity level (Fig. 3).

### Altered gravity influences actin stress fiber organization

**Fig. 4:**
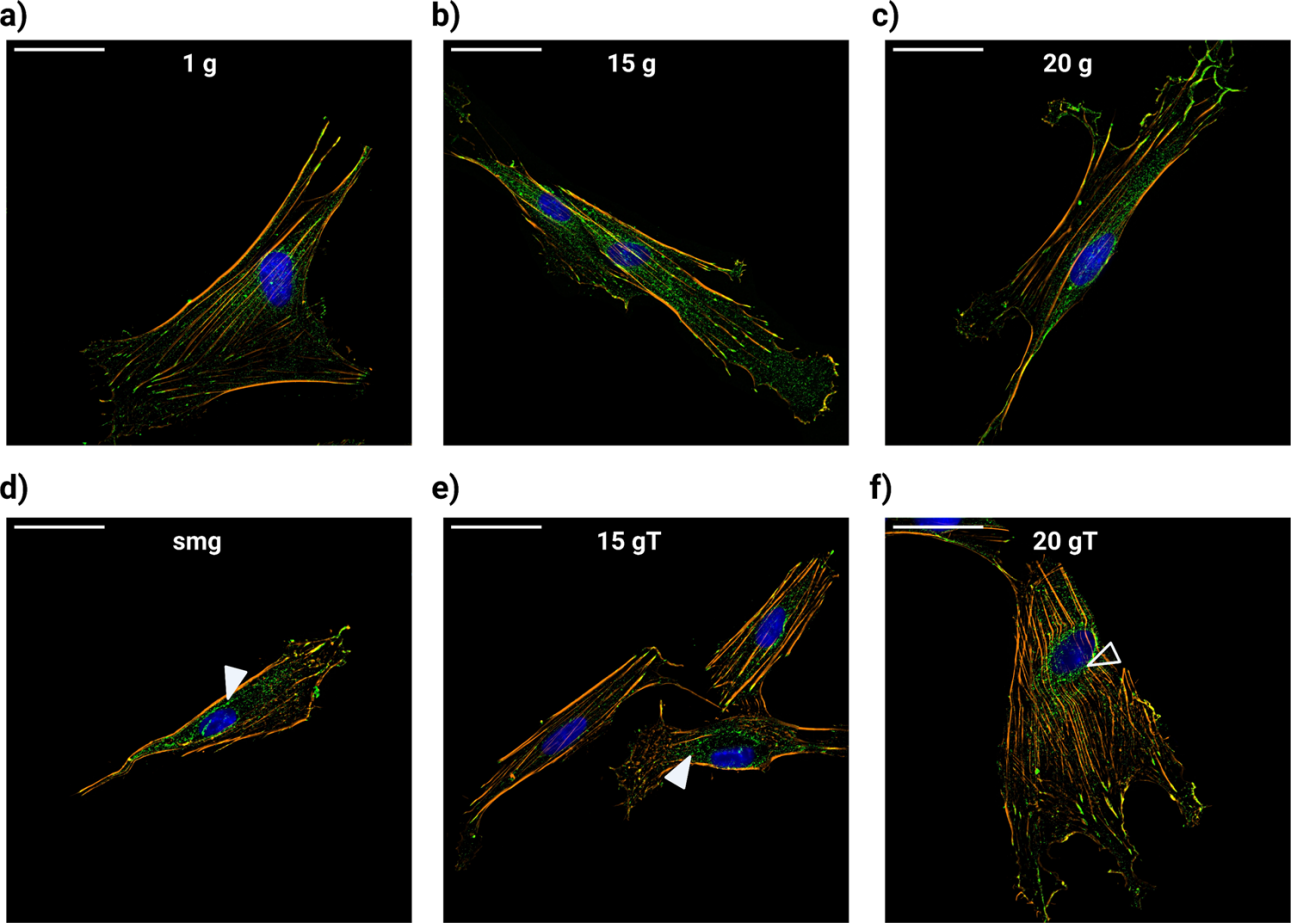
Immunofluorescence staining of phalloidin for actin structures (orange), vinculin for focal adhesions (green), and DAPI for cell nucleus (blue). **a** 1 × *g* control fibroblasts. **b** Fibroblasts exposed to 15 × g hypergravity. **c** Fibroblasts exposed to 20 × g hypergravity. **d** Fibroblasts exposed to simulated microgravity. **e** Fibroblasts exposed to 15 × g hypergravity transitions. **f** Fibroblasts exposed to 20 × *g* hypergravity transitions. Solid white arrows show loss of perinuclear actin fibers in fibroblasts exposed to simulated microgravity (either with or without hypergravity exposure). Open arrow shows bending of perinuclear actin fibers surrounding the nucleus. Scale bar = 50 µm. smg = simulated microgravity, 15 × *g* T and 20 × *g* T are hypergravity transition groups.

Fibroblasts can express a variety of actin stress fiber structures. More thick fibers (mostly associated with myofibroblasts) are essential for contractility and the remodeling of ECM but limit the motility of these cells. On the other hand, dynamic remodeling of the actin cytoskeleton results in less organized thinner fiber bundles which allow cell movement^16^. We investigated if changes in gravity level influenced the organization of the actin cytoskeleton. Cells exposed to simulated microgravity, either solely or with intermitted hypergravity exposure, lacked perinuclear actin fibers in some cells (Fig. 4b, d, solid arrows), which was not observed in the other experimental groups.

**Fig. 5:**
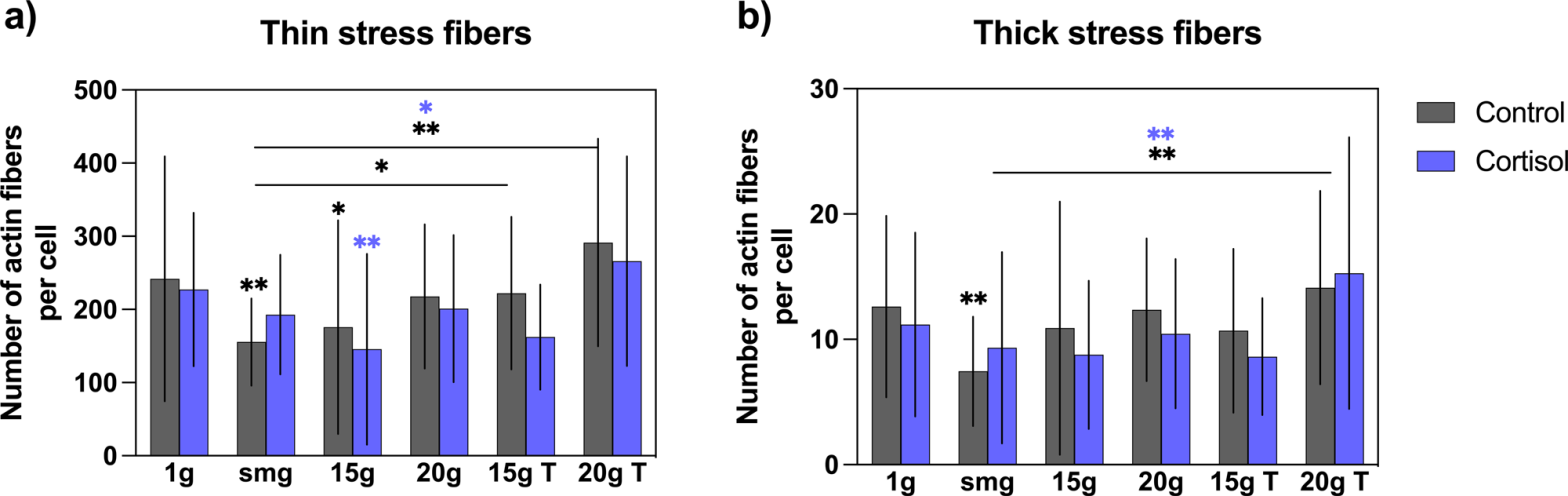
Effect of altered gravity on the number of actin stress fibers per cell. Simulated microgravity significantly reduced both the amount of thin (panel **a**) and thick (panel **b**) actin stress fibers (*P* = 0.0027, *n* = 31 for thin, and *P* = 0.0035, *n* = 31 for thick fibers) which was counteracted by intermitted hypergravity exposure. smg = simulated microgravity. 15 × *g* T and 20 × *g* T are hypergravity transition groups. Error bars show standard deviations. *P < 0.05, **P < 0.005, ***P < 0.001. Statistical tests included ANOVA followed by post hoc analysis (see "Statistical analyses”). Asterisks without line show significance level compared to 1 × *g* control or cortisol exposed cells.

Next, we quantified the amount of thick (4–15 pixels in width)- and thin (1–3 pixels in width) stress fibers per cell. For both thick and thin actin stress fibers per cell, a significant main effect of gravity level was found (thick: *P* = 0.0002, *F* (5, 398) = 4.954, *η*² = 5.8, thin: *P* < 0.0001, *F* (5, 398) = 8.146, *η*² = 9.2). Hypergravity exposure at 15 × *g* significantly reduced total amount of thin actin stress fibers (Fig. 5a, *P* = 0.011, *n* = 47), whereas there was no effect of hypergravity found for the number of thick actin fibers (Fig. 5b). A significant reduction in both thick and thin stress fibers was found in the simulated microgravity group (*P* = 0.0027, *n* = 31 for thin and *P* = 0.0035, *n* = 31 for thick fibers).

However, this effect of simulated microgravity on the amount of stress fibers was not seen in cortisol-exposed cells. Fibroblasts in the 20 × *g* transition group showed an increased amount of both thin (*P* = 0.0001, *n* = 19) and thick (*P* = 0.002, *n* = 19) stress fibers compared to fibroblasts that were exposed to simulated microgravity only, indicating a recovering effect of the intermitted hypergravity exposure on actin stress fibers. This was observed in both control and cortisol-exposed fibroblasts (Fig. 5).

### Gravity transition increased the occurrence of altered nuclear morphology

**Fig. 6:**
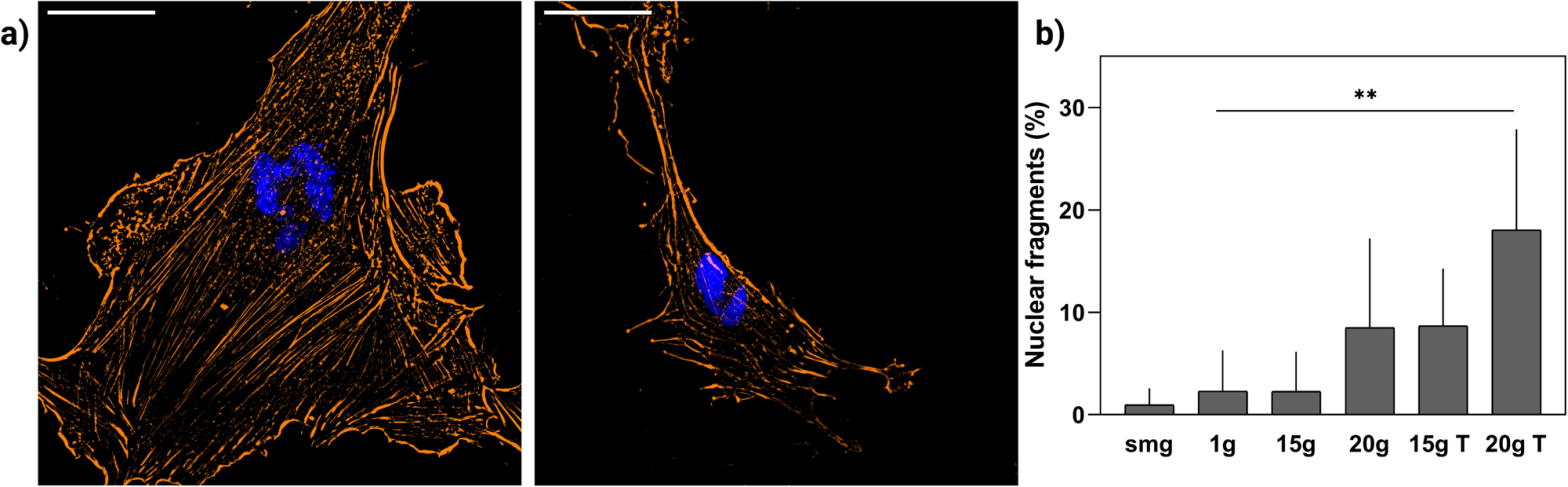
Increased nuclear fragments in fibroblasts exposed to 20 × *g* transitions. **a** Immunocytochemical staining of phaloidin for actin filaments (orange) and DAPI for cell nuclei). Cells showed increase in nuclear fragments (left panel) as well as rupture of the nuclear envelope (right panel). **b** Significant increase in the percentage of nuclear fragments in fibroblasts exposed to 20 × *g* transition compared to 1 g controls (*P* = 0.004, *n* = 102). Error bars show standard deviation. smg = simulated microgravity. 15 × *g* T and 20 × *g* T are hypergravity transition groups. **P < 0.005. Statistical tests included ANOVA followed by post hoc analysis (see "Statistical analyses”).

Fibroblasts were immunocytochemically stained for cytoskeletal components of actin and vinculin as well as DAPI stain to visualize the nucleus. For each cell, the number of nuclear fragments was counted to obtain percentage values of the total number of fragments per condition. In fibroblasts exposed to simulated microgravity intermitted with a hypergravity transition of 20 × *g*, we found an increased number of nuclei with altered morphology. Nuclei were segmented into two or more connected lobes (Fig. 6a, right panel) or had multiple smaller disconnected nuclear bodies (Fig. 6a, left panel). A significant main effect of gravity (*P* = 0.001, *F* (5, 22) = 6.179, *η*² = 55.8) was found. Cells with a high number of nuclear fragments were often enlarged and irregular shaped, which is in accordance with an apoptotic or senescent phenotype (Fig. 6a).

### Cortisol reduces cytokine expression in fibroblasts cultures

**Fig. 7:**
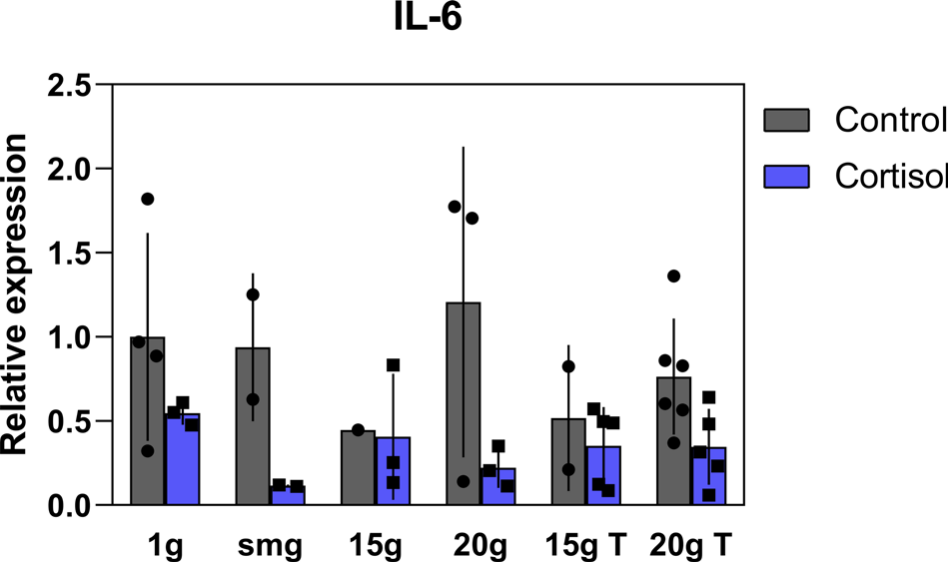
Effect of cortisol and altered gravity on interleukin-6 expression of fibroblasts. Cortisol exposure significantly reduced fibroblast expression of interleukin-6 (IL-6) (*P* = 0.003, *F* (1, 27) = 10.62). Error bars show standard deviations. No effect of gravity could be established. Measures are normalized to total protein content and divided by mean value of 1 × *g* controls to obtain relative values. Statistical tests included ANOVA followed by post hoc analysis (see “Statistical analyses”).

Fibroblasts express a variety of cytokines and growth factors which are crucial for a proper wound-healing process^10^. Pro-inflammatory cytokine interleukin-6 (IL-6) and interleukin-1 receptor antagonist (IL-1RA) regulate the inflammatory response and stimulate fibroblasts to proliferate and remodel the ECM^17,18^. Furthermore, transforming growth factor-beta (TGF-β) plays an important role during the wound-healing process as it induces the differentiation of fibroblasts to myofibroblasts crucial for wound closure and ECM protein expression^10,19^. Here, we investigated if fibroblast expression of cytokine and growth factors were altered after exposure to different gravity levels with or without the addition of cortisol. For IL-6 measures, three outliers were identified and removed from the data. Due to several readings below baseline, total number of measurements was relatively low. Nevertheless, a main effect of cortisol exposure was observed, and cortisol exposure significantly reduced fibroblast expression of IL-6 (*P* = 0.003, *F* (1, 27) = 10.62, *η*² = 21.4) regardless of gravity level (Fig. 7). No effect of gravity on the expression of both cytokine and growth factors was found except for a slightly upregulated TGF-β expression in the 15 × *g* T group (*P* = 0.018, *n* = 3) as compared to 1 × *g* controls (data not shown).

### Cortisol significantly alters ECM expression of dermal fibroblasts

**Fig. 8:**
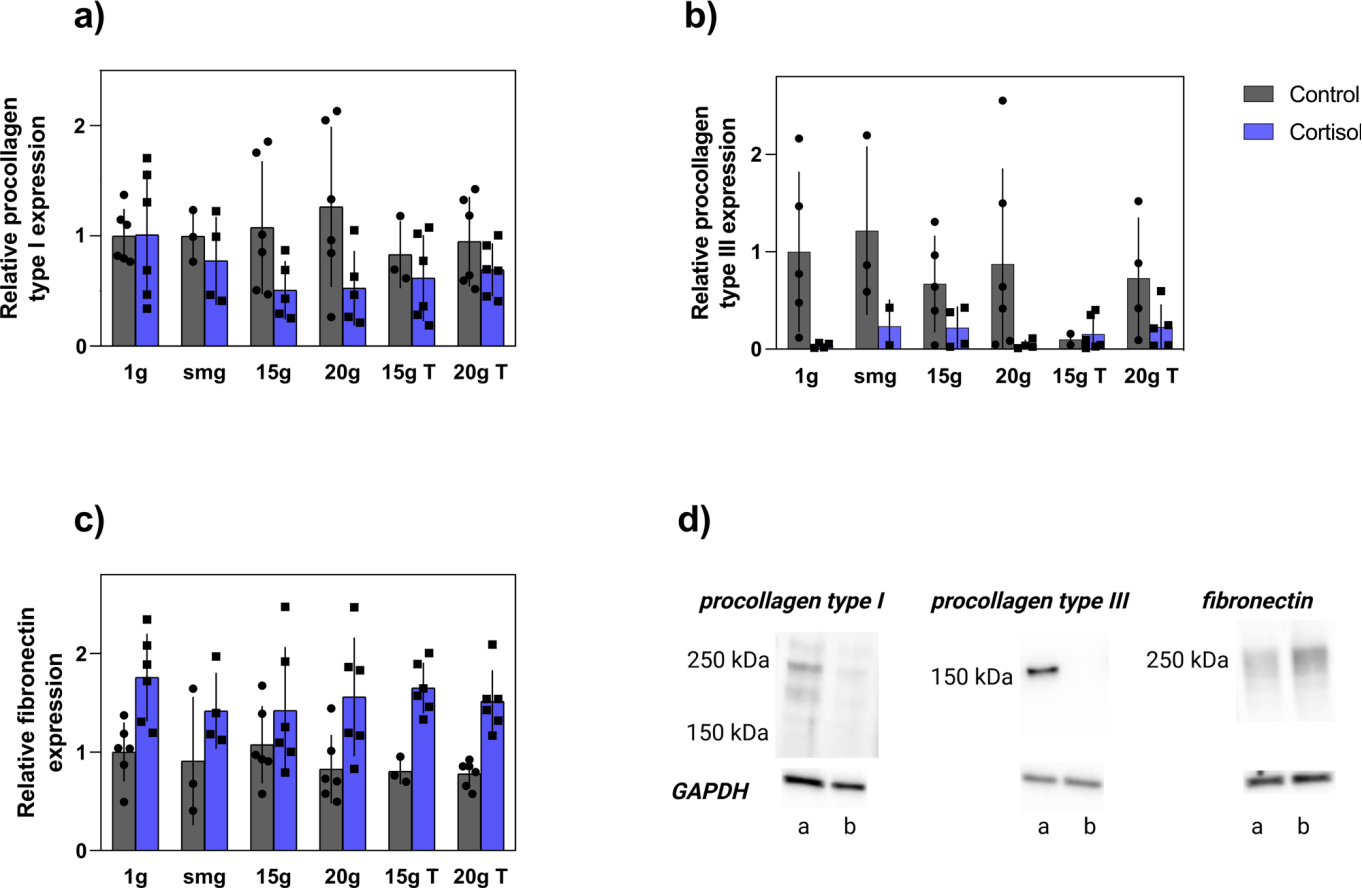
Effect of altered gravity levels and cortisol on fibroblasts expression of ECM proteins. Cortisol exposure significantly reduced expression of procollagen type I (*P* = 0.006, *F* (5, 50) = 8.087) & III (*P* = 0.0007, *F* (5, 38) = 13.45) (**a**, **b**) and increased the expression of fibronectin (*P* < 0.0001, *F* (1, 52) = 37.2) (**c**). Error bars show standard deviations. Statistical tests included ANOVA followed by post hoc analysis (see "Statistical analyses”). **d** Expression of procollagen type I (20 × *g* T) and III and fibronectin (1 × *g*). Line a: control, line b: cortisol. Measures are normalized to total protein content and divided by mean value of 1 × *g* controls to obtain relative values.

Besides the expression of cytokines and growth factors, fibroblasts play an important role in remodeling the connective tissue during wound healing by expressing a number of ECM proteins^10^. To investigate the effect of altered gravity levels and cortisol on fibroblast expression of ECM proteins, we performed western blot analyses for procollagen type I and III as well as fibronectin. Outliers were identified (14 out of 60 for procollagen type III, 2 for procollagen type I) and removed from the data. For all the selected ECM proteins, no effect of altered gravity was found to affect their expression. However, a significant main effect of cortisol was found in all groups. Reduced levels of procollagen type I (α2, *P* = 0.006, *F* (5, 50) = 8.087, *η*² = 12.1, Fig. 8a) and type III (*P* = 0.0007, *F* (5, 38) = 13.45, *η*² = 21.5, Fig. 8b) were found as compared to controls. However, cortisol exposure significantly increased the expression of fibronectin (*P* < 0.0001, *F* (1, 52) = 37.2, *η*² = 38.4, Fig. 8c) when compared to controls.

## Discussion

To better understand the effect of altered gravity levels on fibroblasts’ function related to wound healing, we used an in vitro model to evaluate the role of altered gravity levels, including gravity transitions on several aspects related to the wound-healing process in cultured normal human dermal fibroblasts (NHDF). Furthermore, we exposed fibroblast to cortisol to account for effects of psychological stress, which might interact with altered gravity in the wound-healing process. We found that altered gravity levels mostly affected the migration of fibroblasts as well as their mechanosensitive structures of actin stress fibers and focal adhesions. For the former, intermitted hypergravity transitions increased the number of stress fibers compared to cells exposed to simulated microgravity only. Interestingly, cortisol interacted with and influenced the effect of gravity on most of these endpoints. Besides, we found that gravity transitions induced the occurrence of changes in nuclear morphology. Finally, cortisol exposure mostly affected fibroblast expression of inflammatory cytokine IL-6 as well as extracellular matrix proteins of procollagen type I and III and fibronectin whereas altered gravity did not have a significant effect on these endpoints.

During our experiments, fibroblasts that had been exposed to 20 × *g*, 15 × *g* and 20 transitions showed reduced migration capacity compared to 1 × *g* controls. At continuous exposure to 15 × *g*, hypergravity did not significantly affect fibroblasts migration capacity. The difference in migration capacity between the 15 × *g* and 15 × *g* transition groups, suggests this cellular function is more sensitive to exposure to different gravitational transitions. Of interest is the finding where altered gravity levels did not seem to affect migration in fibroblasts that had been exposed to cortisol as well. Yet, this interaction effect was not significant.

To better understand the observed effects of reduced migration of fibroblasts in altered gravity levels, we further investigated mechanosensitive cytoskeletal components of focal adhesion (FA) complexes and actin stress fibers^20,21^. Actin filaments are linked to the ECM through a combination of transmembrane integrins and protein complexes including vinculin, talin and zyxin^15^. This linkage allows cells to sense and adapt to environmental changes in forces such as ECM stiffness and have an important role in fibroblast motility during wound healing^22^.

In our study, altered gravity levels including simulated microgravity and hypergravity at 15 × *g* and 20 × *g* as well as exposure to 15 × *g* and 20 × g gravity transitions, reduced the number of vinculin spots per cell. However, when cells were exposed to cortisol as well, no effect of simulated microgravity on the number of vinculin spots was observed. Reduction in vinculin has also been found in juvenile NHDF that were cultured on the RPM for three days^23^. Furthermore, hypergravity exposure at 20 × *g* for 48 h induced changes in vinculin anchor points shape of fibroblasts, which induced a decreased adhesion of cells to the substratum^24^. Vinculin knock-out cells have reduced migration capability which was shown as slower and shorter cell displacement. Fibroblasts deprived of vinculin further show reduced directional migration related to wound healing^25^. The finding of the reduced amount of vinculin spots, as well as decreased migration of fibroblasts exposed to 20 × *g* hypergravity and 15 × *g* and 20 × *g* gravity transitions further supports the notion of the gravity-sensitive nature of vinculin which may influence cell migration capacity at altered g-levels.

In our study, simulated microgravity reduced the amount of both thin and thick stress fibers (Fig. 5). In addition, cells that had been exposed to 15 × *g* had fewer thin actin stress fibers as compared to controls (Fig. 5a). Yet, these groups did not show significant differences in their migration capacity compared to the 1 × *g* control group (Fig. 2). Furthermore, cells exposed to continuous 20 × *g* hypergravity had no observable changes in the number of actin stress fibers (Fig. 5). Interestingly, this group had a reduced migration capacity (Fig. 2). The same notion holds for cells exposed to 15 × *g* and 20 × *g* transitions where the number of stress fibers (both thin and thick) did not significantly differ from the 1 × *g* control group. However, compared to simulated microgravity exposure, these groups had an increased number of actin stress fibers (Fig. 5). In these gravity transition groups, the migration of these cells was also hampered (Fig. 2). These findings suggest that the remodeling of the actin cytoskeleton, as observed by the reduction in number of fibers in the simulated microgravity group and the 15 × *g* group, enabled the cells to migrate better compared to groups where no remodeling of the cytoskeleton was observed. Moreover, an increase in the number of stress fibers in the gravity transition groups as compared to the simulated microgravity group was linked to reduced migration capacity. A possible explanation could be that the increase in the number of fibers fixed the cells to the substratum, thereby limiting their motility. On the contrary, dynamic remodeling of the actin cytoskeleton supported fibroblasts motility.

Although we did not find a significant delay in migration in simulated microgravity-exposed cells, in a previous study, a delayed migration in fibroblasts exposed to simulated microgravity was found^11,12^. A possible explanation for this seemingly contradiction might be found in our study design, as we were only able to measure migration of cells after fixation at a specific time point. For this reason, we may have missed subtle dynamic changes in the migration of fibroblasts. To better understand the effects of simulated microgravity on fibroblast migration capacity, it could be of interest to repeat these studies, possibly using live-cell imaging.

Contrary to our findings of lower total number of thin stress fibers in fibroblasts exposed to 15 × *g*, an increase in the total number and thickness of actin stress fibers in osteoblasts exposed up to 4 × *g* of hypergravity was found^26^. In a similar fashion, 3 × *g* hypergravity exposure induced the formation of actin stress fibers in endothelial cells and enhanced their migration capacity^27^. The response of the actin cytoskeleton to hypergravity is both time and force-dependent, as indicated by Costa-Almeida and colleagues who exposed tendon cells to different hypergravity levels. At *g*-levels up to 10 × *g*, total amount of stress fibers decreased, whereas the opposite happened in the higher *g*-level exposures (15 × *g* and 20 × *g*). However, this effect was only observed in cells exposed to 4 h of hypergravity and the reverse was shown in longer durations of hypergravity exposure (16 h)^28^. The differences between our findings and the described literature may indicate a cell-specific formation of stress fibers in reaction to hypergravity. Another possible explanation might also be that the fixation of cells at a single time point may have caused to overlook discrete dynamic responses of the actin cytoskeleton to the hypergravity exposure. To better understand the effects of hypergravity on the remodeling of the cytoskeleton and migration capacity of fibroblasts, future studies should include more hypergravity levels as well as different time points. An interesting development here is the integration of live-cell imaging platform on the DLR human centrifuge, which has been used to track dynamics of F-actin rearrangement in murine astrocytes^29^. Such systems could be used to investigate the rearrangement of actin cytoskeleton in fibroblasts as well at different gravity levels.

In addition, it would be interesting to investigate the fibroblast-to-myofibroblast transition. In the results presented in this paper, fibroblasts exposed to 20 × *g* transitions had an increase in the number of thick stress fibers compared to cells exposed to simulated microgravity only. Thick actin fibers containing alpha-smooth muscle actin (α-SMA) are associated with myofibroblasts. Myofibroblasts play a crucial role in the wound-healing process and have a sophisticated interaction with their microenvironment. Factors such as ECM stiffness and hypoxia can influence the function of these cells and thereby the process of wound healing^30^. To better understand the transition from fibroblast to myofibroblast after exposure to different gravity levels, it would therefore be interesting for future studies to investigate the expressions of α-SMA in these cells.

Finally, our results showed an increase in cells without perinuclear actin fibers in fibroblasts that were exposed to simulated microgravity, either solely or intermittent with hypergravity (Fig. 4b, d). Perinuclear actin fibers are connected to the ECM through focal adhesion and extend above the nucleus where they are linked to the nuclear envelope. Perinuclear actin fibers form an important role during cell migration by reorienting the nucleus, thereby promoting polarization and directional migration^31^. Furthermore, nuclear confinement by perinuclear actin fibers can induce nuclear envelope rupture^32^. The loss of perinuclear actin caps in fibroblasts exposed to simulated microgravity together with the significant increase in actin stress fibers observed in the 20 × *g* transition groups, might explain our finding of increased incidence of altered nuclear morphology induced by gravity transitions (Fig. 6). Nuclear envelope rupture as result of compression by perinuclear actin structures can lead to chromatin fragmentation and DNA damage leading to genomic instability with increased risk of cancer progression^33^. During future space missions to the Moon and Mars, astronauts will be more frequently exposed to gravity transitions after previously being exposed to microgravity. These transitions from microgravity to hypergravity, together with the increased risk associated with ionizing radiation to which astronauts are exposed in space, might increase the risk for developing illnesses later in life. This finding highlights the need for future studies to investigate the effects of gravity transitions on nuclear morphology and how this may lead to altered cellular function or possibly apoptosis. In addition, investigation into how exposure to different gravity fields might interact with ionizing radiation to induce DNA damage and affect the DNA damage repair response could provide more insights into risks associated with spaceflight-associated exposure to ionizing radiation.

In the next paragraphs, we discuss the observation of cortisol effects on cytokine and ECM expression. The expression of pro-inflammatory cytokines during the early stages after wounding is essential for a proper wound-healing process. These cytokines stimulate a variety of processes including fibroblast proliferation, migration and synthesis of extracellular matrix proteins^17^. In our study, cortisol exposure significantly reduced the expression of IL-6. Besides, significant reduced synthesis of both procollagen type I and III were observed in fibroblasts that had been exposed to cortisol compared to control. During the last phase of wound healing, fibroblasts secrete and deposit first the quickly produced collagen type III, which over time, is replaced by the collagen type I, and provides the wound with more strength^34^. IL-6 is an important factor during the wound-healing process including stimulating the synthesis of collagens in fibroblasts^35,36^. Reduced levels of this cytokine due to cortisol exposure likely contributed to the reduced synthesis of these collagens in our experiments. Besides, cortisol administration has been shown to drastically reduce the synthesis of collagen type I and III in rats and humans^37,38^, further supporting our findings of cortisol-induced reduction of procollagen type I and III expression in fibroblasts.

In addition to reduced levels of procollagen, cortisol significantly upregulated the expression of fibronectin. During wound healing, cellular fibronectin excreted by endothelial cells and fibroblasts assemble into a dense network within the granulation tissue. This process is crucial as cells use the established matrix to adhere and migrate into the wound site^39^. In comparison to our findings, treatment of fibroblasts cultures with cortisol upregulated the expression of fibronectin in other studies as well^40–42^. Excessive deposition of fibronectin has been found in keloid tissue which contributes to abnormal wound healing where a nodular scar is formed that extends the size of the original wound. Fibroblasts derived from keloid tissue show upregulated expression of fibronectin similar to that of glucocorticoid-induced increase in fibronectin expression^42^. However, related to the keloid phenotype, the increase in fibronectin observed after cortisol treatment in our experiments is contradictory to that of the decreased collagen expression, as keloid fibroblasts show increased excretion of collagen type I. Therefore, our findings further support the notion of distinct mechanisms underlying upregulation of fibronectin expression in fibroblasts as described in Babu et al.^42^.

Besides the role of the fibroblast to synthesize and deposit collagens, remodeling of the ECM during wound healing involves degradation of provisional matrix by matrix metalloproteinases (MMPs)^10,43,44^. Fibroblasts expression of MMPs has previously been shown to be upregulated after exposure to 20 × *g* of hypergravity for 8 days^45^. In addition, altered expression of MMPs have also been observed in keratinocytes and fibroblasts exposed to simulated microgravity^23,46^. In space, changes in dermal matrix proteins have both been observed in astronauts^5,7^. In addition, alteration of expression of genes encoding MMPs and increase collagen turnover have been found in space-flown mice^47,48^. To better understand the role of different gravitational levels as well as the interaction with cortisol on dermal matrix integrity, it is therefore of interest for future studies to further investigate both the synthesis, deposition and degradation (through means of MMPs) of collagens.

In this work, we have used in vitro cultures of damaged human dermal fibroblasts to investigate the effects of exposure to altered gravity conditions and increased levels of psychological stress on their function relevant for wound healing. However, the skin is a complex organ and its function for maintaining a proper barrier is regulated by a tight interaction between the nervous-, endocrine, and immune system^49–51^. As a result, fibroblasts’ function in vivo is regulated by a plethora of different cell types. The 2D monocultures that were used in these experiments are unable to mimic this complexity. Therefore, these experiments should be repeated with more complex in vitro and in vivo models.

Taken together, our findings show that altered gravity levels and psychological stress both impact functions of fibroblasts which are relevant for proper wound healing. Hypergravity significantly hampered fibroblast migration, which is an important factor of fibroblasts during the wound-healing process. The observed reduced migration could be explained by the reduction of the mechanosensitive structures of focal adhesion complexes. On the other hand, remodeling of the actin cytoskeleton as shown by reduced number of actin stress fibers, seemed to improve cell motility in simulated microgravity. Interestingly, cells that had been exposed to gravity transitions showed an increase in the amount of stress fibers compared to cells in the simulated microgravity group which possibly contributed to a limited motility of these cells. Cortisol mostly impacted the expression of pro-inflammatory cytokines in fibroblasts, which may have implication for during the inflammatory phase of wound healing. Furthermore, fibroblast synthesis of ECM proteins was significantly affected after exposure to cortisol. This could have further implications during the remodeling phase of wound healing. Interestingly, exposure to 20 × *g* hypergravity transitions (20 × *g* T) induced changes in nuclear morphology, indicating the sensitivity of nuclei to micro-to-hypergravity transitions which might impose additional risks for chromosomal instability. These results provide better understanding of the sensitivity of dermal fibroblasts to different spaceflight stressors of altered gravity and psychological stress. The findings of this study can be implemented in future studies aiming to counteract observed changes in wound healing. For example, the effect of the administration of exogenous cytokines and growth factors with the aim to restore the cortisol-induced reduction in the expression of pro-inflammatory cytokines can be investigated. Growth factors act as chemoattractant and mitogen for fibroblasts and stimulate the expression of ECM proteins by these cells^17^. Therefore, the effect of exogenous administration of cytokines and growth factors on countering the reduced collagen expression after exposure to cortisol, as well as the migration capacity of fibroblasts under altered gravity, can also be investigated. The results of such studies will contribute to better protection of astronauts on future long-duration space missions and will be relevant for patients experiencing difficulties in wound healing on Earth.

## Methodology

### Cell culture

Primary Normal Human Dermal Fibroblasts (NHDF, PromoCell, C-12302) obtained from one donor (33-year-old female), were cultured in Gibco Dulbecco’s Modified Eagles Medium containing GlutaMAX^TM^ (DMEM, Gibco, 10566016) media supplemented with 10% fetal bovine serum (FBS, Gibco, 10500064) and 0.25% penicillin–streptomycin (Sigma-Aldrich, P4333). Cells were incubated at 37 °C and 5% CO_2_ in a humidified incubator until they reached 80-90% confluency upon which they were passaged using 0.05% Trypsin-EDTA (Gibco, 25300062) for dissociation.

### Experimental procedures

To study the effects of exposure to altered gravity levels in combination with high levels of the stress hormone cortisol on wound-healing capacities of fibroblasts, an in vitro wound-healing model was used. NHDF cells were seeded inside SlideFlask (Thermo Scientific Nunc Lab-Tek, 170920) or T12.5 flasks (Corning) at densities of ~9000 cells/cm^2^ using 10% serum DMEM medium and left to attach overnight. After cellular attachment, cells were washed with PBS and incubated with medium containing stress hormones or a control vehicle for 48 h. Subsequently, for both flask types, cell monolayers were scratched using a 1 mL pipette tip to damage the cells and create an injury. Flasks were then filled with CO_2_ calibrated culture medium containing stress hormones or a control vehicle and airtight sealed using polymer-integrated sterilized caps followed by placement in the altered gravity condition for 24 h including simulated microgravity, 15 × *g*, 20 × *g*, 15 × *g*, and 20 × *g* gravity transitions, and a 1 × *g* control group. Afterward, cells in SlideFlasks were rinsed with PBS and fixed using 10% formalin solution (Sigma-Aldrich, HT5014). Cells grown in T12.5 flasks were used for the collection of cell lysates (as described in “Extracellular matrix protein expression”). Measures related to fibroblasts’ function of wound healing were obtained, including cellular migration, cytokine and growth factor expressions, and ECM expression. An overview of experimental conditions can be found in Table 1. Figure 1 represents an overview of the sequential order of exposure. For each group, a total of six technical replicates were used.

**Table 1:** Overview of experimental conditions.

Stress conditions	
	Control vehicle (PBS)
	Hydrocortisone 1 µM (HC)
Altered gravity conditions	
	1 × *g* control (1 × *g*)
	Simulated microgravity (smg)
	15 × *g*
	20 × *g*
	15 × *g* Transition (15 × *g* T)
	(14 h smg, 6 h 15 × *g*, 4 h smg)
	20 × *g* Transition (20 × *g* T)
	(14 h smg, 6 h 20 × *g*, 4 h smg)

### Exposure to cortisol

A stock solution of 1 mg/mL (2.76 mM) of hydrocortisone (HC, Sigma-Aldrich, H0888) was made in 96% ethanol. This stock solution was further dissolved in phosphate-buffered saline (PBS) to obtain a concentration of 100 µM. A control vehicle of 96% ethanol dissolved in PBS was used for the control condition. These HC and control solution were 1/100 diluted in cell culture media to obtain a final HC concentration of 1 µM.

### Exposure to altered gravity

A research project proposal was accepted and selected by the ESA Academy to take part in the 2021 Spin Your Thesis! Campaign which granted two and a half days of experimental time on the Large Diameter Centrifuge as well as the Random Positioning Machines (RPM) located in ESA-ESTEC. Simulated microgravity conditions were created using an RPM^52^. Cell culture flasks were filled with media and sealed airtight. To prevent air to be trapped inside caps and prevent gas exchange which can create air bubbles during simulated microgravity exposure, caps were sealed using a polymer (SYLGARD 184 Silicone Elastomer, Dow, 01673921). Cells were exposed to simulated microgravity for 24 h. Flasks showing air bubbles after simulated microgravity exposure were discarded.

Hypergravity was obtained by spinning samples inside the Large Diameter Centrifuge (LDC)^53^ located at European Space Research and Technology Centre (ESTEC). Cell culture flasks were placed inside non-humidified incubators at 37 °C which were placed inside the gondola of the LDC. Control samples were placed at a 1 × *g* environment in the center gondola of the LDC.

Two hypergravity transition groups were included which consisted of intermittent exposure of fibroblasts previously kept in simulated microgravity for 14 h, after which they were exposed to 15 × *g* (15 × *g* T) or 20 × *g* (20 × *g* T) for 6 h. After the hypergravity transition, flasks were placed back in the simulated microgravity environment for 4 h.

In addition, to investigate the effects of hypergravity alone, samples were exposed to 15 × *g* and 20 × *g* for 24 h. This time point was chosen based on preliminary data where migration of fibroblasts under normal conditions shows relative wound closure higher than 50%. Previous studies have shown significant effects of hypergravity on fibroblasts at levels of 10, 20, 24, and 50 × *g*^24,54^. Moreover, exposing fibroblasts to lower levels of hypergravity (below 15 × *g*) did not induce significant changes in cytoskeleton and ECM organization^24^. For this reason, hypergravity levels of 15 × *g* and 20 × *g* were chosen.

### Cytokine and growth factor expression

Fibroblast expression of pro-inflammatory cytokines and growth factors and were quantified in lysates (see “Extracellular matrix protein expression” for a description of how lysates were collected) with Multiplex immunoassay (Interleukin-6 (IL-6), Interleukin-1 receptor agonist (IL-1RA), Luminex Discovery Assay, LXSAHM-03, R&D systems) and ELISA assays (transforming growth factor-beta (TGF-β1), Human TGF beta 1 ELISA kit, ab108912, Abcam) following manufactures instructions. Analyses was performed on Luminex (MAGPIX system with xPONENT 4.3 software (Luminex Corporation. A DiaSorin Company)) instrument for the Multiplex immune assay (analysis performed with Belysa® Immunoassay Curve Fitting Software (Merck, KGaA) and on the absorbance 96 Plate Reader (Enzo Life Sciences, Inc) measured at 450 nm and background subtraction at 570 nm (analysis performed with Byonoy software (Byonoy GmbH). Total protein quantification was done with bicinchoninic acid assay (Sigma-Aldrich), and these data were used for normalization of the data.

### In vitro scratch wound assay

An in vitro scratch wound assay was used to evaluate cellular movement and investigate the effect of the experimental conditions on wound closure. After scratch creation, cells were washed twice with PBS before adding medium containing HC or a control vehicle and 1% FBS to reduce the effect of cell proliferation on wound closure. The scratch was observed under a microscope (Leica, ×5 objective) for capturing baseline images. After 24 h of altered gravity exposure, cells were rinsed with PBS and fixed using 10% Formalin solution (Sigma-Aldrich, HT5014) and images of the scratch were captured. For each replicate, three images were captured at the same position for both the baseline and 24-h time point. Due to air bubble formation several flaskettes were discarded resulting in five replicates for the HC smg group, four in the control vehicle 15 × *g* T, two in the HC 15 × *g* T, and two in the control vehicle 15 × *g* T groups.

### In vitro scratch wound assay—image analysis

Matlab High-Throughput Microscopy Wound Healing Tool^55^ was used to determine the wound area in each picture. Different thresholds were tested to optimize masking of the wound area. Masked images were then visually inspected, images with faulty mask or overcrowding of cells at the wound edge were excluded from the data. Relative wound closure was measured by the following formula:

1 $$\frac{t\left(0\right)-t(24)}{t(0)}* 100 \%$$

Where *t*(0) is the pixel count in the wound area directly after scratching and *t*(24) is the pixel count in the wound area after 24 h of altered gravity exposure.

### Cytoskeletal remodeling

Immunocytochemistry was used for visualization of actin stress fibers and vinculin focal adhesions. Cells were exposed to the different stress medium and gravity conditions as described in “Experimental procedures”. Three out of six replicates were processed for immunocytochemistry analyses of the cytoskeleton. Fixed cells were incubated in PBS containing 0.1% Triton-X 100 and 3% BSA for 5 min at room temperature and washed with Tris buffered saline containing 0.1% Tween (TBS-T). Blocking was performed with 5% goat serum in Tris-NaCl-blocking buffer (TNB) for one hour at room temperature. Primary antibody to stain vinculin focal adhesions (mouse monoclonal anti-vinculin (7f9), Santa Cruz, sc-73614) at a dilution of 1/500 in TNB was added and samples were incubated overnight at 4 °C. Afterwards, samples were washed in TBS followed by incubation with the secondary antibody (Alexa Fluor goat-anti-mouse 488, Invitrogen A11001, 1/500) and Phalloidin 594 (actin stress fibers) TRITC (Invitrogen A12381) in TNB for two hours at room temperature in the dark. Counterstain was carried out using DAPI (1 µg/mL). Immunofluorescence-stained cells were visualized using a Nikon Ti Eclipse inverted widefield fluorescence microscope (Nikon Instruments) with ×60 objective and immersion oil. z-stacks of 11 images were taken 0.5 µm apart.

### Cytoskeletal remodeling—image analysis

Image processing was performed in Fiji v1.53c (http://fiji.sc). Preprocessing steps for images containing vinculin cytoskeleton included summation of z-stack images followed by deconvolution with DeconvolutionLab2^56^ using the build-in algorithm Richardson-Lucy at 25 iterations. A theoretical point-spread function was computed using software package PSFGenerator^57^ using the Born and Wolf model for diffraction with refractive index n_i_ = 1.51 nm, numerical aperture NA = 1.4 and wavelength *λ* = 558 nm for the FITC fluorophore. Next, images were background subtracted with rolling ball radius of 25 pixels. The number of vinculin spots (focal adhesion) per cell were counted using Fiji macro processing pipeline as described in De Vos et al*.*^58^. Focal adhesion images were enhanced with a Laplacian operator and thresholded following the triangle algorithm. Spots smaller than 150 pixels were discarded as they do not represent mature functioning focal adhesions^15^.

Summation of z-stack was also performed on images of actin stress fibers. Images were then processed using freely available toolbox FSegment^59^. This automated image processing algorithm included preprocessing (depending on specific fiber width) and segmentation of thin and thick fibers followed by analyses of fiber length, width, orientation and intensity. Per cell, the total number of thick and thin fibers were quantified.

### Extracellular matrix protein expression

Expression of extracellular matrix proteins of procollagen type I & III as well as fibronectin was determined through a western blot assay. After gap creation, flasks were completely filled with serum free medium containing stress hormones or a control vehicle and airtight sealed using polymer-integrated sterilized caps followed by placement in the altered gravity condition. Due to air bubble formation, several flasks had to be discarded which resulted in three replicates for the control vehicle smg group, four for the HC smg, and four for the control vehicle 15 × *g* T group.

After exposure to altered gravity conditions, cells were dissociated using 0.25% trypsin-EDTA (Gibco, 25200072). Cell pellets were washed using ice-cold PBS and re-suspended in ice-cold Radioimmunopreciptation assay (RIPA) buffer (Pierce, 89901). To increase yields, Tissue Lyser II (Quiagen) P2 30.01/s was used for 2 min. Cell lysates were centrifuged at 14,000 × *g* for 10 min at 4 °C to pellet down the cell debris and supernatant was collected into a fresh Eppendorf tube. Cell lysates were stored at −80 °C.

Total protein quantification was performed using Bicinchonic acid assay (Sigma-Aldrich). Cell pellets were diluted in milliQ to a concentration of 5 µg/µL. In all, 4× Lammli buffer with β-mercaptoethanol (1/10) were added to the protein samples. Samples used for measuring the expression of fibronectin were incubated at 95 °C for 10 min, samples for measuring expression of collagen type I and III were not heated. Protein samples were then subjected to a 4–15% Criterion TGX Stain-Free Precast Gel, followed by transfer to a nitrocellulose membrane for collagen type I and fibronectin, and a polyvinylidene difluoride (PVDF) membrane for collagen type III. Unspecific binding sites were blocked in 5% milk powder in TBS-T for 2 h at room temperature followed by incubation with primary antibody overnight (rabbit polyclonal to Collagen I (1/1000, Abcam, ab34710) for collagen type I, rabbit monoclonal recombinant anti-collagen III (1/2000, Abcam, ab184993) for collagen type III, and mouse monoclonal anti-fibronectin (1/1000, Sigma-Aldrich, F0916) for fibronectin). In addition, for all blots, primary antibodies mouse monoclonal to glyceraldehyde 3-phosphate dehydrogenase (GAPDH) were added (1/10,000, Abcam, ab8425) which served as loading control. Primary antibodies were diluted in blocking buffer (5% milk powder in TBS-T) and incubated overnight at 4 °C. After washing in TBS-T, membranes were incubated with secondary horseradish peroxidase (HRP)-conjugated antibody diluted in blocking buffer (1/2000 for collagen type I, 1/10 000 for collagen type III, 1/1000 for fibronectin and 1/1000 for GAPDH). Enhanced chemiluminescence was used for detection of the HRP conjugate (Biorad clarity kit according to the manufacturer’s instructions). Blots were imaged using the Fusion FX (Vilbert). Protein bands for the target proteins were measured using Bio1D (v15.06, Vil-ber Lourmat). Each band was normalized to the loading and internal control.

### Statistical analyses

Statistical analyses were performed in GraphPad Prism version 8.0.0 for Windows (GraphPad Software, San Diego, CA, USA, www.graphpad.com). For each endpoint, outliers were identified using robust regression and outlier removal (ROUT)^60^. To test for significant group differences two-way ANOVA analyses were performed. Data were first tested for normality. Both main and interaction effects of stress and gravity level were tested. Post hoc analyses, using the Benjamini–Krieger–Yekutieli adaptive linear step-up procedure^61^, was then performed on all analyses to identify group differences between each experimental condition. *P* values were corrected for multiple testing using false discovery rate of 5%. For visualization purposes, all graphs are shown relative to 1 × *g* controls.

### Reporting summary

Further information on research design is available in the Nature Research Reporting Summary linked to this article.

## Supplementary information

Reporting Summary

## Data Availability

Data are available upon request.
