# Supplementary material for: Gravitational effects on fibroblasts’ function in relation to wound healing
Source: NPJ Microgravity. 2023 Jun 21;9:48. doi: 10.1038/s41526-023-00286-z (PMC10284925; doi:10.1038/s41526-023-00286-z)
Supplement: Supplementary file 1 — Reporting Summary [file 41526_2023_286_MOESM1_ESM.pdf]

nature portfolio

Corresponding author(s): Radstake, W. E.

Last updated by author(s): Nov 2, 2022

Reporting Summary

Nature Portfolio wishes to improve the reproducibility of the work that we publish. This form provides structure for consistency and transparency in reporting. For further information on Nature Portfolio policies, see our Editorial Policies and the Editorial Policy Checklist.

Statistics

For all statistical analyses, confirm that the following items are present in the figure legend, table legend, main text, or Methods section.

n/a	Confirmed	
☐	☒	The exact sample size (*n*) for each experimental group/condition, given as a discrete number and unit of measurement
☐	☒	A statement on whether measurements were taken from distinct samples or whether the same sample was measured repeatedly
☐	☒	The statistical test(s) used AND whether they are one- or two-sided<br>*Only common tests should be described solely by name; describe more complex techniques in the Methods section.*
☒	☐	A description of all covariates tested
☐	☒	A description of any assumptions or corrections, such as tests of normality and adjustment for multiple comparisons
☐	☒	A full description of the statistical parameters including central tendency (e.g. means) or other basic estimates (e.g. regression coefficient) AND variation (e.g. standard deviation) or associated estimates of uncertainty (e.g. confidence intervals)
☐	☒	For null hypothesis testing, the test statistic (e.g. *F*, *t*, *r*) with confidence intervals, effect sizes, degrees of freedom and *P* value noted<br>*Give P values as exact values whenever suitable.*
☒	☐	For Bayesian analysis, information on the choice of priors and Markov chain Monte Carlo settings
☒	☐	For hierarchical and complex designs, identification of the appropriate level for tests and full reporting of outcomes
☐	☒	Estimates of effect sizes (e.g. Cohen's *d*, Pearson's *r*), indicating how they were calculated

Our web collection on statistics for biologists contains articles on many of the points above.

Software and code

Policy information about availability of computer code

Data collection

data collection of cytokine expression was performed using Luminex MAGPIX system with xPONENT 4.3 software and Byonoy software
analysis of cytokine expression was performed with Belysa Immunoassay Curve Fitting Software
Matlab high-throughput Microscopy Wound Healing Tool was used to determine the wound area
image processing of immunocytochemistry microscopy images was performed using FIJI v1.53c
western blots were imaged using Fusing FX (Vilbert), protein bands for the target proteins were measured using the Bio1D v15.06

Data analysis

statistical analysis were performed in GraphPad Prism v 8.0.0 for Windows

For manuscripts utilizing custom algorithms or software that are central to the research but not yet described in published literature, software must be made available to editors and reviewers. We strongly encourage code deposition in a community repository (e.g. GitHub). See the Nature Portfolio guidelines for submitting code & software for further information.

Data

Policy information about availability of data

All manuscripts must include a data availability statement. This statement should provide the following information, where applicable:

- Accession codes, unique identifiers, or web links for publicly available datasets
- A description of any restrictions on data availability
- For clinical datasets or third party data, please ensure that the statement adheres to our policy

data available upon request

Human research participants

Policy information about studies involving human research participants and Sex and Gender in Research.

Reporting on sex and gender	NA
Population characteristics	NA
Recruitment	NA
Ethics oversight	NA

Note that full information on the approval of the study protocol must also be provided in the manuscript.

Field-specific reporting

Please select the one below that is the best fit for your research. If you are not sure, read the appropriate sections before making your selection.

☒ Life sciences ☐ Behavioural & social sciences ☐ Ecological, evolutionary & environmental sciences

For a reference copy of the document with all sections, see nature.com/documents/nr-reporting-summary-flat.pdf

Life sciences study design

All studies must disclose on these points even when the disclosure is negative.

Sample size	sample sizes determination was based on acquisition of preliminary data, and risk mitigation.
Data exclusions	samples containing airbubbles after exposure to simulated microgravity were discarded<br>scratch images with faulty masking were excluded from the data<br>outliers were identified using robust regression and outlier removal
Replication	given the nature of the experiments, ie. using exclusive research facility not normally accessible, these experiments were performed once.<br>sufficient sample sizes were therefore used to obtain statistical meaningful data.
Randomization	samples were randomly assigned to experimental groups.
Blinding	NA

Reporting for specific materials, systems and methods

We require information from authors about some types of materials, experimental systems and methods used in many studies. Here, indicate whether each material, system or method listed is relevant to your study. If you are not sure if a list item applies to your research, read the appropriate section before selecting a response.

Materials & experimental systems

n/a	Involved in the study
☐	☒ Antibodies
☐	☒ Eukaryotic cell lines
☒	☐ Palaeontology and archaeology
☒	☐ Animals and other organisms
☒	☐ Clinical data
☒	☐ Dual use research of concern

Methods

n/a	Involved in the study
☒	☐ ChIP-seq
☒	☐ Flow cytometry
☒	☐ MRI-based neuroimaging

Antibodies

Antibodies used

rabbit polyclonal to collagen I, Abcam, ab34710
rabbit monoclonal recombinant anti-collagen III, Abcam, ab184993
mouse monoclonal anti-fibronectin, Sigma-Aldrich, F0916
mouse monoclonal to GAPDH, Abcam, ab8425

Validation

ab34710 - immunogen: full length native protein corresponding to human collagen I aa 1-1464. Collagen type I from human and bovine placenta. Suitable for Western blot.
ab184993 - species reactivity: reacts with mouse, rat, human. suitable for Western blot of human skin lysates and whole cell lysates
F0916 - tested for Western blot with human forsekin fibroblasts cells
ab8425 - immunogen: full length native protein corresponding to GAPDH, reacts with mouse, rat, human. Suitable for Western blot.

Eukaryotic cell lines

Policy information about cell lines and Sex and Gender in Research

Cell line source(s)

primary normal human dermal fibroblasts, female, 33 years old, caucasian

Authentication

CD90 positive (flow cytometry)

Mycoplasma contamination

tested negative for micoplasma

Commonly misidentified lines
(See ICLAC register)

NA
